# Liver Metastases of Unknown Primary: Malignant Melanoma

**DOI:** 10.1155/2014/131708

**Published:** 2014-07-20

**Authors:** Ozgur Bostanci, Kinyas Kartal, Muharrem Battal

**Affiliations:** Department of General Surgery, Sisli Etfal Training and Research Hospital, Istanbul 34371, Turkey

## Abstract

According to the National Cancer Institute's (NCI) data, the increase in the number of patients diagnosed with malignant melanoma was found to be at a higher rate than the current increase in all other types of cancer (Jemal et al., 2008). Early diagnosis, appropriate surgical treatment, and chemotherapy have positive impacts on the course of the disease but despite these developments on the treatment, current prognosis of metastatic malignant melanoma prognosis is still extremely poor. Life expectancy in patients with metastatic disease is between 2 and 8 months. The 5-year disease-free survival rate is identified in only 5% of the patients (Leong, 2003) (Kirkwood et al., 1996). In this study, we try to report a patient with metastatic malignant melanoma and give recent informations about the liver metastases of malignant melanoma.

## 1. Case Report

**Figure 1:**
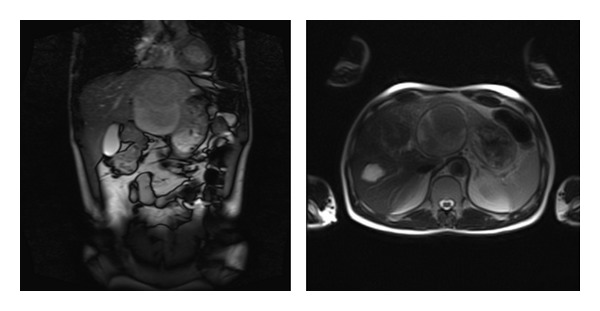
Magnetic resonance imaging sections.

A 59-year-old male without any known health problems was admitted to Sisli Hamidiye Etfal Training and Research Hospital General Surgery Department with a complaint of right upper quadrant abdominal pain and a weight loss of 8 kg in one month period. There were no special features at his medical history. On physical examination, there was a mild right upper quadrant abdominal tenderness on deep palpation. Abdominal ultrasound revealed a mass with 130 × 74 mm diameters, originating from the left lobe of the liver extending to the midline, with multiple thick septas and intense cystic lesions containing echogenic areas identified. Also another 3 cm cystic mass with similar ultrasonographic features was detected at the posterior right lobe of the liver. Magnetic resonance imaging (MRI) was performed for the patient. According to the MRI findings, there was a cystic lesion in the left lobe of the liver with a diameter of 11.5 cm at the widest spot. In the first plan the mass was thought to be a degenerated hemorrhagic adenoma but a metastatic tumor was unable to rule out. MRI images are shown in Figure 1. Upper and lower gastrointestinal endoscopy was performed but no primary tumor was detected.

Laboratory analyses were in normal range while CA19-9 was 29.69 U/mL. Because of the presence of a malignant disease which cannot be ruled out, it was decided that a surgical excision should be performed.

According to the intraoperatively assessment, 10 cm mass was identified in the left lobe of the liver and excised by performing left hepatic lobectomy. The patient was hospitalised for recovery for 4 days and then discharged.

**Figure 2:**
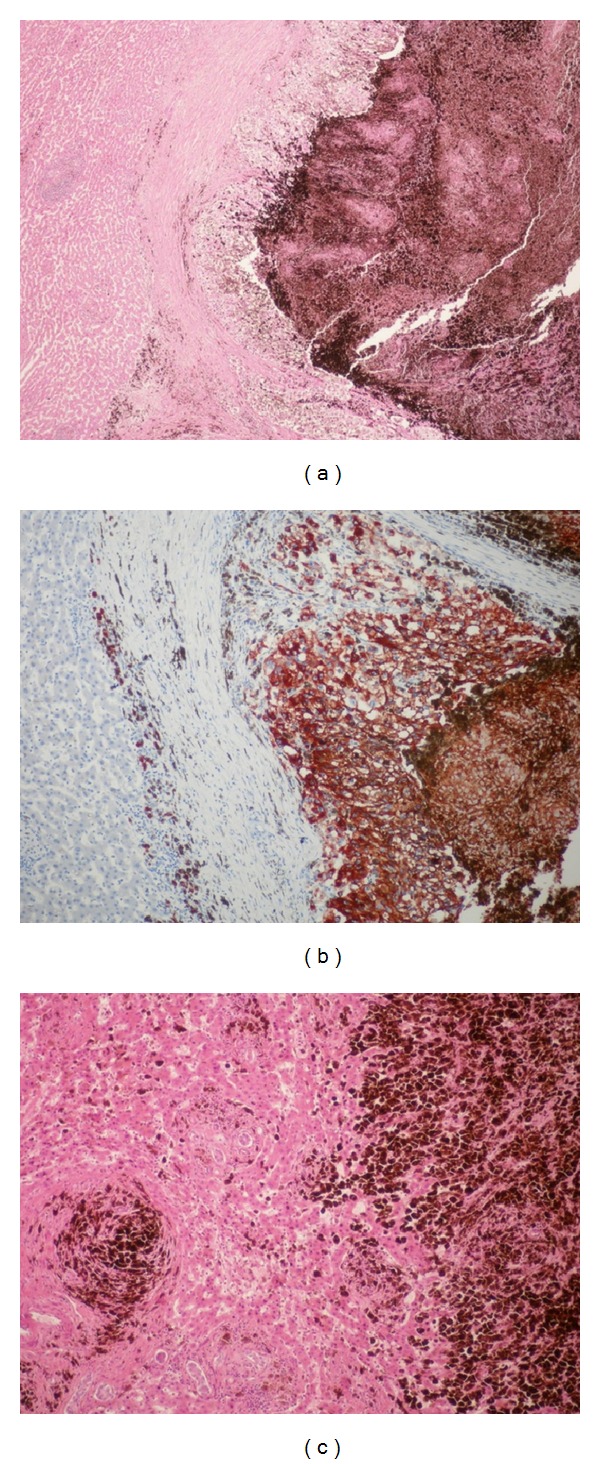
(a) Malignant melanoma, containing melanin pigment in the cytoplasms. Large, pleomorphic, vesicular nuclei and large clear cytoplasm with HMB45 positive reaction. Liver parenchyma in the surrounding, 4 × 10 MR. (b) Malignant melanoma infiltration containing melanin pigment in the cytoplasms. Large, pleomorphic, vesicular nuclei and large clear cytoplasm with atypic cells. Liver parenchyma in the surrounding. H × E, 4 × 10 MR. (c) Malignant melanoma, containing melanin pigment in the cytoplasms, recessive cellular details, infiltrating to the surrounding liver parenchyma. H × E, 10 × 10 MR.

Pathological evaluation showed a 7 × 6 cm cystic, dark brown mass in the 13 × 9 cm liver tissue obtained from left hepatectomy. According to immunohistochemical consideration HMB45, S100 and pCEA were strongly positive; CK8, CK18, CD34, and Glypican 3 were negative for the tumor (Figure 2). In light of the present findings, the lesion has been considered as the liver metastasis of malignant melanoma.

## 2. Discussion

According to the National Cancer Institute's (NCI) data, the increase in the number of patients diagnosed with malignant melanoma was found to be at a higher rate than the current increase in all other types of cancer [1]. Early diagnosis, appropriate surgical treatment, and chemotherapy have positive impacts on the course of the disease but despite these developments on the treatment, current prognosis of metastatic malignant melanoma prognosis is still extremely poor. Life expectancy in patients with metastatic disease is between 2 and 8 months. The 5-year disease-free survival rate is identified in only 5% of the patients [2, 3].

Malignant melanoma is one of the most common tumor which metastases to the gastrointestinal tract [4]. Malignant melanoma modaly metastatsis to lymph nodes (73.6). The second organ that malignant melanoma is metastasis is the lung (71.3%). Liver (58.3%), brain (54.6%), bone (48.6%), and adrenal glands (46.8%) are the other solid organs that malignant melanoma metastases [5]. Memorial Sloan Kettering Cancer Center conducted an autopsy series of malignant melanoma. According to that study, malignant melanoma most commonly metastasizes to the liver in the gastrointestinal tract [6].

Metastatic melanoma of unknown primary was first published in 1963 by Das Gupta et al. [7]. Later studies pointed out that 2% and 6% of all malignant melanoma patients were consisting of metastatic tumors of unknown primary. 10–15% of these patients are thought to be* amelanotic melanoma* patients. Although there is a conflict in the medical literature, it is supposed that the prognosis of metastatic malignant melanoma with unknown primary is similar to melanoma with primary known tumors. Life expectancy of patients with stage IV disease does not exceed more than 9 months [8, 9].

The treatment of early stage malignant melanoma is surgical excision with 1-2 cm tumor-free surgical margins and sentinel lymph node sampling. If sentinel lymph node is evaluated as positive, radical lymphadenectomy is needed to be added to the surgical procedure. There are studies indicating that radiotherapy improves the survival of patients with four or more metastatic lymph nodes [10, 11].

American Joint Committee on Cancer (AJCC) has started to examine the metastatic malignant melanoma cases in three subgroups. Malignant melanoma with skin, subcutaneous tissues, or distant lymph node metastasis were classified as M1a; cases with lung metastases were called M1b and metastasis of other solid organs was classified as M1c [12]. Patients in stage M1a have a life expectancy of 10 to 18 months. It has been evaluated that if radical surgical procedures are implemented to these patients, the life expectancy is extended up to 50 months [13, 14]. After surgical interventions, 5-year survival rate increases from 5% to 29% in patients with M1b disease [15].

According to the AJCC classification, M1c stage differs from M1a and M1b. The common organs that malignant melanoma usually metastasizes are brain, gastrointestinal tract, and liver, respectively. It is identified that 40% of the patients with clinical signs and symptoms of neurological system had brain metastases. In these cases even with surgical interventions or radiotherapy the average life expectancy does not exceed 12 months [16].

Life expectancy of the malignant melanoma with gastrointestinal tract or liver metastases is virtually 6–9 months. This ratio is exceeding 46–48 months after radical surgical interventions performed [17–19].

## 3. Conclusion

Within all of the cancer types, malignant melanoma is the most likely cancer that can metastasize to solid organs. The most important factor in the prognosis of malignant melanoma is the stage of the disease. In every stage of the disease there is a high risk of metastasis and recurrence. Although the metastatic malignant melanoma has a very poor prognosis, recent studies show that radical surgical treatments when combined with chemoradiotherapy provide a significant increase in the life expectancy of these patients.
